# Supplementary material for: Resolving the full spectrum of human genome variation using Linked-Reads
Source: Genome Res. 2019 Apr;29(4):635–45. doi: 10.1101/gr.234443.118 (PMC6442396; doi:10.1101/gr.234443.118)
Supplement: Supplemental Material [file supp_gr.234443.118_Supplemental_longranger-2.2.zip › longranger-2.2.tar.bz2!/lr2.2-public/lib/stan/cmdstan/stan/doc/api/html/agrad_2fwd_2functions_2primitive__value_8hpp_source.html]

Stan: src/stan/agrad/fwd/functions/primitive\_value.hpp Source File


|  |  |
| --- | --- |
|  | Stan  2.5.0  probability, sampling & optimization |


- Main Page
- Related Pages
- Namespaces
- Classes
- Files

- File List
- File Members

All Classes Namespaces Files Functions Variables Typedefs Enumerations Enumerator Friends Macros Pages

- src
- stan
- agrad
- fwd
- functions

primitive\_value.hpp

Go to the documentation of this file.

1 #ifndef STAN\_\_AGRAD\_\_FWD\_\_FUNCTIONS\_\_PRIMITIVE\_VALUE\_HPP

2 #define STAN\_\_AGRAD\_\_FWD\_\_FUNCTIONS\_\_PRIMITIVE\_VALUE\_HPP

3

4 #include <stan/agrad/fwd/fvar.hpp>

5 #include <stan/math/functions/primitive\_value.hpp>

6

7 namespace stan {

8

9  namespace agrad {

10

11

21  template <typename T>

22  inline double primitive\_value(const fvar<T>& v) {

23  using stan::math::primitive\_value;

24  return primitive\_value(v.val\_);

25  }

26

27

28  }

29

30 }

31

32 #endif

stan::agrad::fvar

**Definition:** fvar.hpp:13

fvar.hpp

stan::agrad::primitive\_value

double primitive\_value(const fvar< T > &v)

Return the primitive value of the specified forward-mode autodiff variable.

**Definition:** primitive\_value.hpp:22

primitive\_value.hpp

stan::math::primitive\_value

boost::enable\_if< boost::is\_arithmetic< T >, T >::type primitive\_value(T x)

Return the value of the specified arithmetic argument unmodified with its own declared type...

**Definition:** primitive\_value.hpp:30

stan::agrad::fvar::val\_

T val\_

**Definition:** fvar.hpp:15


---

[ Stan Home Page ]

*© 2011–2014,
Stan Development Team.*
